# Identification of an antibody‐based immunoassay for measuring direct target binding of RIPK1 inhibitors in cells and tissues

**DOI:** 10.1002/prp2.377

**Published:** 2017-12-06

**Authors:** Joshua N. Finger, Jean‐Marie Brusq, Nino Campobasso, Michael N. Cook, Jennifer Deutsch, Heather Haag, Philip A. Harris, Earl L. Jenkins, Devika Joglekar, John D. Lich, Sean Maguire, Rakesh Nagilla, Elizabeth J. Rivera, Helen Sun, Bartholomew J. Votta, John Bertin, Peter J. Gough

**Affiliations:** ^1^ Pattern Recognition Receptor DPU GlaxoSmithKline Collegeville PA USA; ^2^ Structural and Biophysical Sciences GlaxoSmithKline Collegeville PA USA; ^3^ Integrated Biological Platform Sciences GlaxoSmithKline Collegeville PA USA

**Keywords:** Benzoxazepinone, pharmacokinetics/pharmacodynamics, RIPK1, TEAR1, tissue target engagement, TNF

## Abstract

Therapies that suppress RIPK1 kinase activity are emerging as promising therapeutic agents for the treatment of multiple inflammatory disorders. The ability to directly measure drug binding of a RIPK1 inhibitor to its target is critical for providing insight into pharmacokinetics, pharmacodynamics, safety and clinical efficacy, especially for a first‐in‐class small‐molecule inhibitor where the mechanism has yet to be explored. Here, we report a novel method for measuring drug binding to RIPK1 protein in cells and tissues. This TEAR1 (Target Engagement Assessment for RIPK 1) assay is a pair of immunoassays developed on the principle of competition, whereby a first molecule (ie, drug) prevents the binding of a second molecule (ie, antibody) to the target protein. Using the TEAR1 assay, we have validated the direct binding of specific RIPK1 inhibitors in cells, blood and tissues following treatment with benzoxazepinone (BOAz) RIPK1 inhibitors. The TEAR1 assay is a valuable tool for facilitating the clinical development of the lead RIPK1 clinical candidate compound, GSK2982772, as a first‐in‐class RIPK1 inhibitor for the treatment of inflammatory disease.

**Abbreviation**
BOAz: Benzoxazepinone
BTK: Bruton's tyrosine kinase
ECL: Electrochemiluminescent
ELISA: Enzyme‐linked immunosorbent assay
FasL: Fas ligand
GSK2267064: GSK'064
GSK2882481: GSK'481
GSK2982772: GSK'772
GSK3011253: GSK'253
HDX‐MS: Hydrogen deuterium exchange‐mass spectrometry
PK: Pharmacokinetics
RIPK1: Receptor‐interacting serine/threonine‐protein kinase 1
TE: Target Engagement
TEAR1: Target engagement Assessment for RIPK1
TLR3: Toll‐like receptor 3
TLR4: Toll‐like receptor 4
TNF: Tumor necrosis factor
TRAIL: TNF‐related apoptosis‐inducing ligand

## 1 INTRODUCTION

Receptor‐interacting serine/threonine‐protein kinase 1 (RIPK1) has emerged as a critical kinase that has been shown to regulate inflammatory pathways through both scaffolding and kinase‐specific functions.^1^ RIPK1 is most notable for its regulatory role in the tumor necrosis factor (TNF) receptor pathway, which must be tightly regulated to maintain tissue homeostasis. If dysregulated, the TNF receptor pathway may result in spontaneous and robust inflammation.^7^, ^34^ Recent work has shown that RIPK1 kinase activity can drive this inflammation through directly regulating necroptosis and proinflammatory cytokine production.^4^, ^5^, ^11^, ^14^ The regulatory role of RIPK1 is not limited to only TNF receptor signaling, but is a critical driver of inflammation downstream of several other receptor pathways, including FasL, TRAIL, TLR3, and TLR4.^6^, ^23^, ^25^ Recent literature describing the use of selective RIPK1 inhibitors and RIPK1 kinase‐dead knock‐in mice in preclinical models have highlighted the pathogenic role for RIPK1 kinase activity, particularly in diseases associated with aberrant TNF receptor signaling.^3^, ^19^, ^36^ Hence, we believe that blocking this pathway with RIPK1 small‐molecule inhibitors has the potential to result in a broad therapeutic application in multiple inflammatory diseases including psoriasis, ulcerative colitis, and rheumatoid arthritis.

A series of RIPK1 inhibitors was first discovered using a phenotypic cell screen to identify compounds that block necroptotic cell death.^9^, ^10^ We have recently reported on a number of potent inhibitors of RIPK1 kinase activity^2^, ^20^, ^21^ An initial DNA‐encoded library screen yielded the benzodiazepinone (BOAz) series of potent and highly selective RIPK1 kinase inhibitors.^20^ Optimization of this series identified GSK2982772 which has nanomolar potency, exquisite selectivity and an excellent preclinical pharmacokinetic and developability profile leading to its selection for clinical development as a first‐in‐class small‐molecule inhibitor for the treatment of inflammatory disease.^22^

As with any new area of therapeutic intervention, it is not only critical to have a high‐quality molecule, but it is also important to have methods to monitor direct target engagement to properly guide dose selection and interpret pharmacodynamic effects and clinical efficacy.^27^ One of the main issues in monitoring drug efficacy during early clinical development is that drug binding is difficult to measure in cells and tissues.^26^ Traditionally, drug efficacy has been monitored indirectly by assessing cellular responses downstream of the target protein. This can be rather simple if the target protein has a physiological function that is directly tied to alterations in circulating factors such as cytokines or chemokines in the blood. This becomes increasingly difficult, however, when measurement of drug efficacy is required at the tissue site of action where protein functions may not be easily measured.

The evaluation of target engagement at the cellular level is quite challenging as there are relatively few established methods that translate across multiple protein targets. One methodology with potential to translate across multiple discovery programs utilizes an ELISA‐based immunoassay, whereby unoccupied target protein is captured and quantified. A recent work describes such an assay whereby target engagement of Bruton's tyrosine kinase (BTK) using a covalent inhibitor, CC‐292, was assessed by incubating cell or tissue homogenates with a second covalent BTK inhibitor chemically‐linked to biotin, then capturing the biotinylated probe‐BTK complex on a streptavidin plate.^13^

Here, we describe a novel immunoassay method for measuring the direct interaction of the BOAz series of RIPK1 inhibitors to RIPK1 protein. The Target Engagement Assessment for RIPK1 (TEAR1) assay is a simple, yet robust pair of immunoassays that was developed based on the principle of competitive inhibition. Under normal (noninhibited) conditions, both immunoassays detect RIPK1 protein. However, when the RIPK1 inhibitor is present, only the TOTAL‐RIPK1 immunoassay is able to detect RIPK1 protein levels. The FREE‐RIPK1 immunoassay is no longer able to detect RIPK1 protein likely due to structural alterations in the RIPK1 activation loop, the region of epitope recognition by the FREE‐RIPK1 antibody. In this article, we describe the identification of the TEAR1 assay and its potential application for monitoring direct target engagement of BOAz‐RIPK1 inhibitors in clinical tissue samples from patients with inflammatory disease.

## 2 MATERIALS AND METHODS

### 2.1 Reagents

RIPK1 inhibitors were developed at GlaxoSmithKline (Collegeville, PA). SMAC mimetic 2′,2‴‐(2,4‐hexadiyne‐1,6‐diyl)bis[1‐[[(2S)‐1‐(N‐methyl‐L‐alanyl‐L‐threonyl)‐2‐pyrrolidinyl]methyl]‐5‐(phenylthio)‐1H‐tetrazole^24^ was made at GlaxoSmithKline (Collegeville). Recombinant human TNF and z‐VAD‐FMK were purchased from R&D systems (Minneapolis, MN). Antibody reagents were purchased from commercial sources: Anti‐RIPK1 antibodies were from Abcam (Cambridge, MA), Cell Signaling (Danvers, MA), and Santa Cruz (Dallas, TX); Anti‐Actin antibody was from BD Biosciences (San Jose, CA).

### 2.2 Animals

Eight healthy male cynomolgus macaques (*Macaca fasicularis*), age 8‐12 years, previously obtained from Charles River BRF (Houston, TX), Covance (Alice, TX), or Mannheimer (Miami, FL) and housed in an AAALACi‐accredited facility at GlaxoSmithKline (Collegeville) were utilized for the study. These monkeys were housed indoors, maintained on a 12:12‐hour light‐dark cycle, fed a standard primate diet (LabDiet Certified Primate Diet 5043) plus a variety of additional fresh foods and foraging each day, and had access to ad libitum water. They were provided with toys and auditory or visual enrichment daily and acclimated to all conscious study procedures.

### 2.3 Antibody screening by immunoassay

HT‐29 cell (American Type Culture Collection, Manassas, VA) lysates were incubated with increasing concentrations of RIPK1 inhibitor, GSK2882481 (GSK'481), GSK3011253 (GSK'253), or GSK2267064 (GSK'064), for 24 hour at 4°C in lysis buffer. Following incubation, lysates were analyzed by immunoassay using mouse anti‐human RIPK1 antibody (Abcam ab72139; 1 μg·mL^−1^) as capture and various rabbit anti‐human RIPK1 antibodies for primary detection (1 μg·mL^−1^ final concentration). Raw electrochemiluminescent (ECL) counts were plotted for single data points. Percent target engagement was determined based on calculated concentrations using a recombinant human RIPK1 protein (ab135220; Abcam) as a standard curve.

### 2.4 TEAR1 immunoassays

Two RIPK1 immunoassays for each TEAR1 assay were performed on lysates produced from cells and tissues using the MULTI‐ARRAY 96‐well small spot plates (Meso‐Scale Diagnostics, Rockville, MD) coated with mouse anti‐human RIPK1 antibody (ab72139; Abcam) at a final concentration of 1 μg·mL^−1^ overnight at 4°C. Following coating, plates were blocked for 1 hour in 5% Bovine Serum Albumin in PBS, then washed with 150 μL of wash buffer (ie, TBS + 0.05%Tween). Experimental samples and RIPK1 standards were incubated for 2 hours at room temperature, and then washed three times with 150 μL of wash buffer. For FREE‐RIPK1, the 3493 antibody (Cell Signaling) was added to plate 1 at a final concentration of 1 μg·mL^−1^ . For TOTAL‐RIPK1, the ab125072 (Abcam) antibody was added to plate 2 at a final concentration of 0.28 μg·mL^−1^ . Both primary detection antibodies were diluted in 1% BSA in PBS containing 0.1% IGEPAL‐630 and incubated on each plate for 1 hour at room temperature, then washed three times with 150 μL of PBS. Anti‐rabbit IgG, SULFO‐tagged detection antibody (Meso‐Scale Diagnostics) was diluted 500‐fold in 1% BSA in PBS containing 0.1% IGEPAL‐630 and incubated on each plate for 1 hour at room temperature, then washed three times with 150 μL of PBS. Read Buffer T with surfactant (Meso‐Scale Diagnostics) was diluted to 1× in water and 150 μL was added to each well. Plates were analyzed on a Sector S600 plate reader (Meso‐Scale Diagnostics). For concentration response experiments, normalized data were analyzed in Graphpad Prism using nonlinear regression and a 4‐parameter fit curve (variable slope) to determine IC_50_ values for each compound. All the data are shown as mean ± SD.

### 2.5 Treatment of HT29 cells with RIPK1 inhibitors

HT29 human colon adenocarcinoma cells (American Type Culture Collection, Manassas, VA) were plated in 24‐well plates at a density of 200,000 cells·cm^−2^ overnight. On the following day, media were removed and replaced with fresh Dulbecco's Minimal Essential Medium (Invitrogen, Carlsbad, CA) supplemented with 10% fetal bovine serum, 100 units·mL^−1^ penicillin and 100 μg·mL^−1^ streptomycin (Invitrogen). RIPK1 inhibitors were prepared as a 10 mmol·L^−1^ stock solution in DMSO (Sigma, St. Louis, MO) and were added to wells for 24 hours incubated at 37°C, 5% CO_2_. Cells were lysed in 1× RIPA buffer (Millipore, Billerica, MA) containing protease inhibitors (Roche Diagnostics, Indianapolis, IN) and phosphatase inhibitors (Roche Diagnostics) for 1 hour on ice, and then frozen. The next day, cell lysates were thawed and separated by centrifugation at 2500*g* for 10 minutes at 4°C to remove cell debris. Homogenates were analyzed by TEAR1 immunoassay. Experimental results are representative of 3 replicate experiments.

### 2.6 Western blotting

Cell lysates (10 μg) were separated on 8% bis‐tris Bolt gels (Invitrogen) following reduction and denaturation. Following transfer to nitrocellulose membranes, blots were blocked in Protein‐Free TBS blocking buffer (ThermoFisher Scientific, Waltham, MA). Primary antibodies were incubated for 2 hours at room temperature in blocking buffer at a final concentration of 1:1000. Blots were washed three times in TBS + 0.05% Tween, followed by incubation with appropriate secondary antibodies. Immunoblots were read on an Odyssey Imager.

### 2.7 Animal procedures for tissue distribution study

Infusion dosing and blood samples without biopsies were accomplished via conscious techniques. All skin biopsies were done after 10 mg·kg^−1^ Ketamine (Ketaved) IM (Vedco, St. Joseph, MO) and isoflurane (Piramal Healthcare Limited, India) anesthesia. Flunixin Meglumine 1 mg·kg^−1^ IM (Phoenix Pharmaceuticals, St. Joseph, MO) analgesia was given once a day on each biopsy day. Two 4 mm punch biopsies were collected from the upper dorsum after clipping and a surgical scrub. Blood samples at time points with biopsies were obtained after anesthesia. Baseline skin punch biopsies were collected from approximately 2 weeks prior to dosing. Animals were infused with GSK'253 (0.12 mg·kg^−1^: 0.03 mg·mL^−1^ in 20% cavitron and 5% DMSO, 4 mL·kg^−1^) for 4 hours via an IV catheter. Dosing solution was clear and colorless, and was filtered through a 0.22 μmol·L^−1^ PES in‐line filter during infusion. Following final blood sample and/or skin biopsy collection and prior to recovery from anesthesia, animals were euthanized with Fatal‐Plus Solution (Vortech, Dearborn, MI) 100‐150 mg·kg^−1^ IV and terminal tissue samples were collected. All tissues were weighed, stored in cryotubes or foil, snap frozen and kept on dry ice until storage at −80°C.

### 2.8 Analytical methods for GSK'253

Analysis of blood samples from study days for GSK'253 was performed using liquid chromatography‐tandem mass spectrometric (LC‐MS/MS) detection. The samples were thawed, blood proteins were precipitated with 200 μL of 95/5 acetonitrile/0.1% aqueous formic acid, containing 200 ng·mL^−1^ of a mass spectral internal standard (ie, Verapamil), and the resulting mixture was vortex‐mixed for 2 minutes followed by centrifugation for 30 minutes at >2500*g*. Analytical calibration standards were prepared in monkey blood; blood proteins were precipitated as described above. A full standard curve consisting of nine different concentrations (ranging from 0.1 to 1000 ng·mL^−1^) was prepared for GSK'253 in monkey blood. Homogenate of each tissue specimen was prepared in appropriate volume of acetonitrile with a homogenizer. The final weight of each homogenate was determined to calculate the actual homogenate weight:tissue weight ratio. 50 μL aliquots of each homogenate were transferred to sample tubes and stored at approximately −80°C until analysis. Control tissue specimens were also collected from a control animal and a homogenate was prepared as described above. The control homogenates were used for preparation of analytical standards. The HPLC system consisted of a Waters Acquity pump, auto sampler, column oven, and online degaser (Waters, Massachusetts, MA). A 2 × 20 mm, 4 μ, Synergi Hydro RP analytical column was used (Phenomenex, Torrance, CA). The mobile phase consisted of a gradient that transitioned linearly from 95% aqueous 0.1% formic acid / 5% acetonitrile to 100% acetonitrile over 1.0 minutes at 750 μL·minutes^−1^ flow rate. A 2.0 μL aliquot of samples were directly injected in to a Sciex triple quadrupole API5000 mass spectrometer (Applied Biosystems, Foster City, California; Software: Analyst 1.6.2) using turbo ion spray source in positive mode followed by multiple‐reaction monitoring. GSK'253 was characterized by the transition of the *m/z* 396.2 parent (M + H) + precursor ion to its *m/z* 204.1 product ion, generated at optimized collision energy at 35V and declustering potential at 110V, respectively. Data were reported as quantitative drug concentrations as determined by standard calibration curve analysis, using a linear fitting of either (1/x) or 1/(x*x) weighted plot of the GSK'253/internal standard peak area ratios vs GSK'253 concentration.

### 2.9 Tissue homogenization

Nonhuman primate tissues were transferred to prefrozen (−80°C) 2.0 mL safe‐lock microcentrifuge tubes (Eppendorf, Hauppauge, NY) containing two 5‐mm stainless steel beads (QIAGEN Inc., Germantown, MD) and maintained on dry ice in a CoolRack M96ID cooling rack (Corning, Corning, NY). Prior to homogenization, homogenization tubes were transferred to a CoolRack M96ID cooling rack maintained on wet ice. RIPA lysis buffer (0.5 mL), diluted to 1× in water and supplemented with protease inhibitors and phosphatase inhibitors, was immediately added to tubes. Tubes were capped tightly, transferred to prechilled 24‐well TissueLyser adaptors, and homogenized in the TissueLyser (QIAGEN Inc., Germantown, MD) for 3 cycles of 3 minutes at 30 Hz. Following homogenization, tissue homogenates were collected briefly by centrifugation (500*g*; 1 minute) and incubated on ice for 1 hour. Following incubation on ice, insoluble debris was removed by centrifugation at 15,000*g* for 10 minutes at 4°C. For blood samples, whole blood (50 μL) was diluted to 10% with 1× RIPA lysis buffer and incubated on ice for 30 minutes followed by centrifugation at 15000*g* for 10 minutes at 4°C. Soluble blood and tissue lysates were analyzed by the TEAR1 immunoassay.

### 2.10 Statisical analysis

Statistical analysis was performed using GraphPad Prism V6.0. Unless otherwise specified, data are presented as mean ± SD. Comparisons were performed with a Student's t test whose values are represented in the figures as *P < .05, **P<.01, and ***P<.001.

### 2.11 Ethics Statement

The animals were housed and maintained in accordance with standards established in the Animal Welfare Act and the Guide for the Care and Use of Laboratory Animals. All studies involving the use of animals were conducted after review and approval by the GlaxoSmithKline (GSK) Institutional Animal Care and Use Committee and in accordance with the GSK Policy on the Care, Welfare and Treatment of Laboratory Animals. All animal procedures were performed under approved IACUC animal use protocol PA0483.

## 3 RESULTS

### 3.1 BOAz‐RIPK1 inhibitors induce unique conformational changes in RIPK1 protein

**Figure 1:**
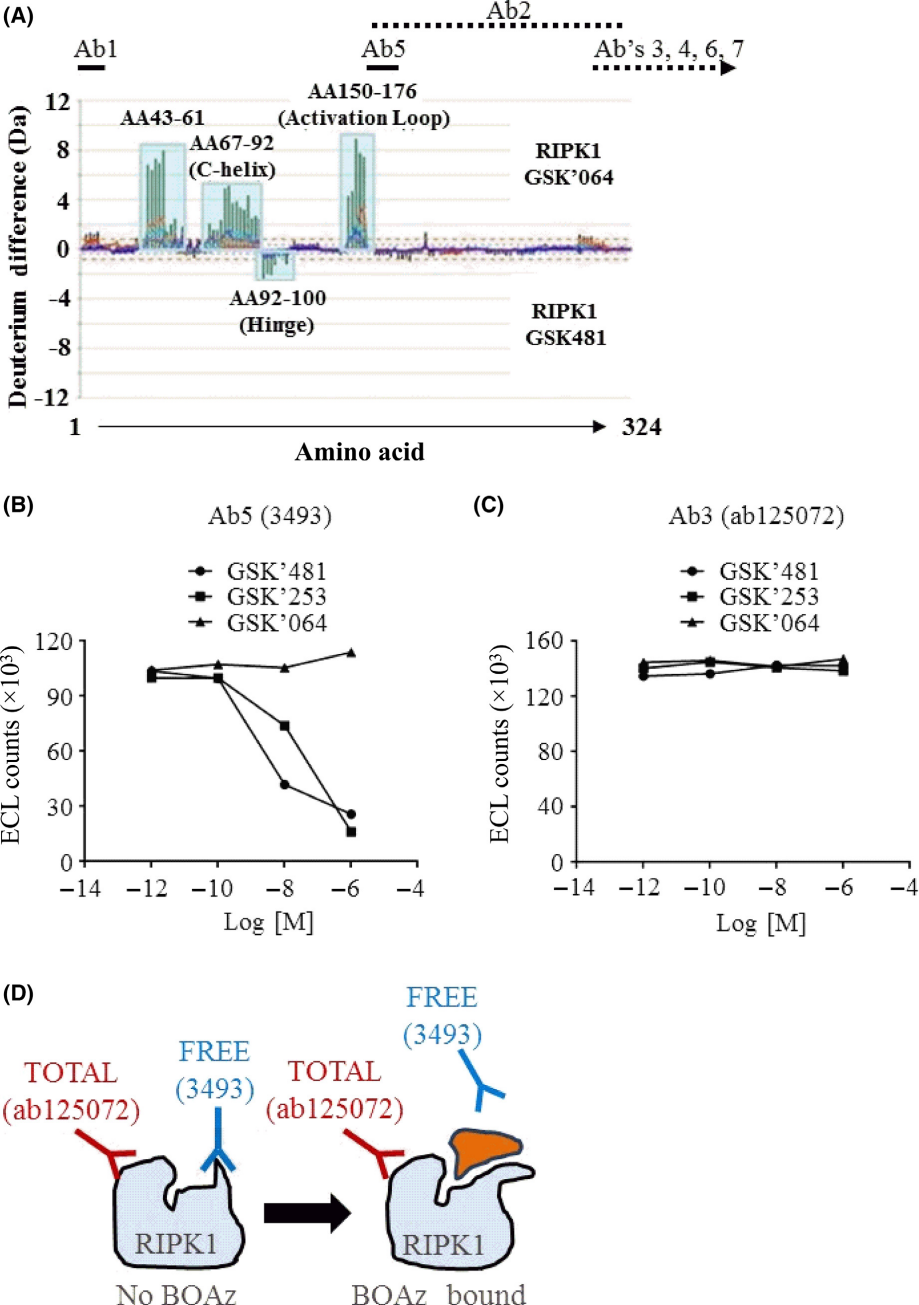
Identification of a target engagement antibody for RIPK1. (A) Comparison of RIPK1 antibodies (Ab1‐Ab7) with changes observed in HDX‐MS experiment between GSK’481‐bound protein and GSK’064‐bound protein. Ab3 is abcam 125072. Ab5 is Cell Signaling 3493. Ab3, Ab4, Ab6, and Ab7 are shown, though they do not overlap with the RIPK1 (1‐324) truncated protein used in HDX‐MS. These antibodies recognize a region at the RIPK1 C‐terminus from AA325‐671. The exact epitopes of these antibodies remain unknown. (B) HT‐29 cell lysates (20 μg) were incubated with GSK’481, GSK’253, or GSK’064 at concentrations ranging from 1 μmol·L^−1^ to 1 pmol·L^−1^ for 24 hours. Cell lysates were analyzed by immunoassay using Abcam ab72139 mouse monoclonal antibody for RIPK1 capture and Ab5 (Cell Signaling 3493) at 1 μg·mL^−1^ final concentration. Raw ECL counts were plotted against log molar concentrations of RIPK1 inhibitor. Samples were screened in single well format. (C) HT‐29 cell lysates (20 μg) were incubated with GSK’481, GSK’253, or GSK’064 at concentrations ranging from 1 μmol·L^−1^ to 1 pmol·L^−1^ for 24 hours. Cell lysates were analyzed by immunoassay using Abcam ab72139 mouse monoclonal antibody for RIPK1 capture and Ab3 (Abcam ab125072) at 1 μg·mL^−1^ final concentration. Raw ECL counts were plotted against log molar concentrations of RIPK1 inhibitor. Samples were screened in single well format. (D) Schematic representation of target engagement model demonstrating “competition” of FREE‐RIPK1 antibody by RIPK1 inhibitors of the BOAZ chemical class. ECL, Electrochemiluminescent

To identify regions of the RIPK1 protein that undergo structural change upon binding of RIPK1 inhibitors, we compared the differential exchange rates of amide hydrogen with deuterium in the RIPK1 protein backbone using hydrogen‐deuterium exchange mass spectrometry (HDX‐MS). There was a marked decrease in deuterium incorporation in RIPK1 protein when the BOAz‐RIPK1 inhibitor, GSK2882481 (GSK'481), was bound vs the traditional hinge‐binding RIPK1 inhibitor, GSK2267064 (GSK'064). As can be seen in Figure 1A, peptide regions near the G‐loop (AA43‐61), C‐helix (AA67‐92), and activation loop (AA150‐176) incorporated more deuterium in the GSK'064 inhibitor‐protein complex than in the GSK'481 inhibitor‐protein complex suggesting that these regions become more protected from solvent when the GSK'481 inhibitor is bound. Additionally, peptide regions containing residues in the hinge region (AA92‐100) incorporated more deuterium in the GSK'481 inhibitor‐protein complex suggesting that these regions become less ordered than in the GSK'064 complex or are potentially blocked by the hinge‐binding GSK'064. These structural changes in RIPK1 protein upon BOAz‐RIPK1 inhibitor binding were confirmed with our recently published cocrystal structure data using GSK'481.^21^

### 3.2 The selective RIPK1 inhibitors, GSK2882481 and GSK3011253, compete with antibody binding to RIPK1 protein

On the basis of the changes observed during our HDX‐MS studies and co‐crystallization efforts, we hypothesized that these changes in RIPK1 structure might be leveraged to monitor compound binding to RIPK1 protein using an immunoassay format with similarity to an assay that was developed for Bruton's tyrosine kinase.^13^ To identify antibodies whereby the antibody binding is potentially altered when a RIPK1 inhibitor is present, we screened commercially available antibodies (Figure 1A; Table S1) in a sandwich immunoassay format using a mouse anti‐human RIPK1 antibody to immunoprecipitate RIPK1 protein. Surprisingly, the detection of RIPK1 protein by the CS3493 antibody (Ab5) following immunoprecipitation was decreased in a concentration‐dependent manner when HT29 cell lysates were incubated with GSK'481 or GSK3011253 (GSK'253), but not with the traditional hinge‐binding GSK'064 (Figure 1B; Figure S1). In comparison, the detection of RIPK1 protein by the ab125072 antibody (Ab3) was unaffected following incubation with all three compounds (Figure 1C). Depicted in Figure 1D, based on these initial findings, antibody CS3493 recognizes the RIPK1 protein within the RIPK1 activation loop in the unbound state, when RIPK1 is “FREE” of the RIPK1 inhibitor. When a BOAz‐RIPK1 inhibitor is bound to RIPK1 protein, the epitope for the CS3493 antibody is no longer accessible for binding. On the other hand, antibody ab125072 (TOTAL) recognizes RIPK1 protein irrespective of inhibitor binding. This assay has been termed the TEAR1 assay.

### 3.3 Engagement of RIPK1 can be detected in cells using the TEAR1 assay

**Figure 2:**
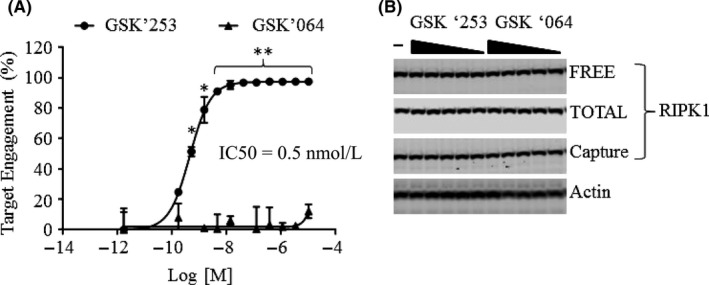
RIPK1 target engagement in HT‐29 cells using the TEAR1 assay. (A) HT29 cells were incubated with either GSK’253 or GSK’064 for 24 hours. Cells lysates (20 μg) were analyzed for RIPK1 target engagement using both FREE‐RIPK1 and TOTAL‐RIPK1 immunoassays. FREE‐RIPK1 levels were normalized to TOTAL‐RIPK1 levels. Data are represented as the percent target engagement ±SD; n = 3 replicates per group. *P < .05, **P < .01, and ***P < .001. (B) HT29 cell lysates (10 μg) was analyzed by western blot for RIPK1 (CS3493, ab125072, and ab72139) and normalized to actin

We sought to perform a similar set of compound experiments in HT29 cells using the TEAR1 assay. We focused on the GSK'253 RIPK1 inhibitor as it has better oral pharmacokinetic properties than GSK'481. As shown in Figure 2A, GSK'253 demonstrated a concentration‐dependent increase in RIPK1 target engagement (IC_50_ = 0.5 nmol·L^−1^). The measurement of FREE‐RIPK1 in the immunoassay was inversely proportional to the concentration of GSK'253, whereas GSK'064 failed to induce any change in antibody binding to RIPK1 protein (Figure S2). Neither GSK'253 nor GSK'064 had any effect on RIPK1 protein expression or degradation in HT29 cells (Figure 2B).

### 3.4 Measuring RIPK1 Target Engagement in cynomolgus monkeys using the TEAR1 assay

**Figure 3:**
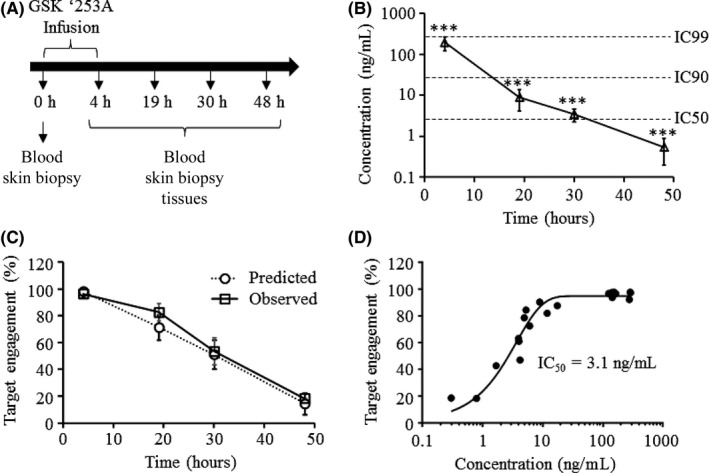
Target engagement of gsk'253 in cynomolgus monkey whole blood. (A) Study design for in vivo evaluation of RIPK1 target engagement. (B) Blood from monkeys (n = 2/group) was collected at various time points following IV administration of 0.12 mg·kg^−1^ GSK’253 and analyzed by LC‐MS/MS. ***P < .001. (C) Comparison of predicted and observed RIPK1 target engagement following IV administration of GSK’253. Predicted target engagement was calculated using the known IC50 of 3.1 ng·mL^−1^ in a monkey whole‐blood challenge assay, assuming a hill slope of 1. Data are represented as the percent target engagement +/‐ SD; n = 2 animals per group. (D) Comparison of GSK’253 PK and in monkey whole blood following IV administration and observed target engagement. Individual measurements are plotted and IC50 was calculated in GraphPad Prism using a nonlinear regression and a 4‐parameter curve fit

As the BOAz class of RIPK1 inhibitors has a consistent species selectivity for inhibition of RIPK1 in primates over nonprimates,^21^ we sought to compare the PK and RIPK1 target engagement in tissues following IV dosing in cynomolgus monkeys. Based on previously determined pharmacokinetic parameters of GSK'253 (data not shown), eight male cynomolgus monkeys (n = 2/group) were dosed with 0.12 mg·kg^−1^ GSK'253 via a 4 hour IV infusion to allow the drug to reach an approximate steady state concentration. For appropriate measurement of target engagement, baseline blood and skin sampling was performed 2 weeks prior to dosing. At the predetermined time points of 4, 19, 30, and 48 hours after the start of IV infusion of GSK'253, blood and skin punch biopsies were collected from each animal under anesthesia (Figure 3A). Following the final biopsy collection, each cohort was euthanized under anesthesia and terminal colon and synovial tissues were collected.

Based on the measured drug concentrations in the blood, RIPK1 target engagement was predicted using the IC_50_ of 3.1 ng·mL^−1^ for GSK'253, as determined in previously established cell‐based assays (data not shown). Immediately after infusion, blood‐drug levels had an observed Cmax of 197 ± 72 ng·mL^−1^ (Figure 3B). Maximal target engagement was predicted to reach 98.3 ± 0.6% based on the observed Cmax with predicted levels decreasing to 14.6 ± 8.1% by 48 hours postinfusion (Figure 3C). Observed RIPK1 target engagement closely matched the predicted target engagement based on drug PK (Figure 3C; Table S2). GSK'253 demonstrated an IC_50_ of 3.1 ng·mL^−1^ matching that which was predicted from cell‐based assays (Figure 3D).

**Figure 4:**
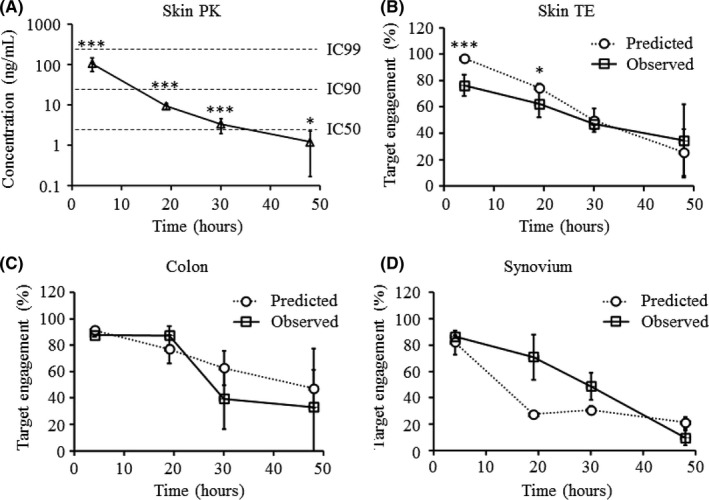
Target engagement of GSK’253 in cynomolgus monkey tissues. (A) Skin biopsy‐drug concentrations at each time point following IV administration of 0.12 mg·kg^−1^ GSK’253. Data are represented mean drug concentration ± SD; n = 2 monkeys per group. *P < .05, **P < .01, and ***P < .001. (B) Comparison of predicted and observed RIPK1 target engagement in skin biopsies following IV administration of GSK’253. Predicted target engagement was calculated using the known IC50 of 3.1 ng·mL^−1^ in a monkey whole‐blood challenge assay, assuming a hill slope of 1. Data are represented as the percent target engagement ± SD; n = 2‐8 animals per group. *P < .05, **P < .01, and ***P < .001. (C) Comparison of predicted and observed RIPK1 target engagement in terminal colon tissue following IV administration of GSK’253. Predicted target engagement was calculated using the known IC50 of 3.1 ng·mL^−1^ in a monkey whole‐blood challenge assay, assuming a hill slope of 1. Data are represented as the percent target engagement ±SD; n = 2 animals per group. (D) Comparison of predicted and observed RIPK1 target engagement in terminal synovium tissue from knee joints following IV administration of GSK’253. Predicted target engagement was calculated using the known IC50 of 3.1 ng·mL^−1^ in a monkey whole‐blood challenge assay, assuming a hill slope of 1. Data are represented as the percent target engagement ±SD; n = 2‐8 animals per group

To better define the utility of the TEAR1 assay in a clinically relevant target tissue associated with psoriasis, we analyzed skin biopsy samples and compared the observed target engagement to predicted target engagement based on tissue PK. Immediately after infusion, drug levels in the skin had an observed Cmax of 108 ± 39 ng·mL^−1^ (Figure 4A, Table S3). Maximal target engagement was predicted to reach 96.9 ± 1.0% based on the observed Cmax with predicted levels decreasing to 25.8 ± 17.9% by 48 hours postinfusion (Figure 4B). Observed target engagement in skin biopsies correlated well with predicted target engagement based on tissue PK (Table S3). At early time points (ie, 4 and 19 hours), target engagement was overestimated based on drug concentrations vs what was measured in the tissue using the TEAR1 assay, whereas at later time points (ie, 30 and 48 hours), predicted and observed levels were similar. These data suggest a time‐dependent transit of the RIPK1 inhibitor to its target protein.

To expand our tissue target engagement analysis beyond skin samples, we evaluated RIPK1 target engagement in colon and synovial tissues, as these tissues are the major sites of inflammation in TNF‐dependent diseases such as ulcerative colitis and rheumatoid arthritis, respectively. Colon and synovial tissue samples were isolated from each cohort (n = 2/cohort) and analyzed using the TEAR1 assay. After infusion, observed drug levels in the colon (Figure 4C and Table S4) and synovium (Figure 4D and Table S5) were similar to the predicted levels based on tissue PK.

## 4 DISCUSSION

RIPK1 has emerged as an significant drug target for the treatment of a number of inflammatory disorders ranging from TNF‐mediated diseases such as ulcerative colitis and multiple sclerosis, to ischemic events such as myocardial infarction and stroke, to acute critical‐care situations such as burn injury, sepsis, and toxic epidermal necrolysis.^12^, ^28^, ^29^, ^31^, ^32^, ^33^, ^35^ We have previously reported on identification of GSK2982772 as a novel, potent, ultraselective inhibitor of RIPK1 22 that has recently completed Phase 1 safety and tolerability studies in healthy volunteers^15^ and is currently being explored in Phase 2a experimental medicine studies for the treatment of psoriasis,^16^ ulcerative colitis,^17^ and rheumatoid arthritis.^18^ As GSK2982772 is the first RIPK1 inhibitor to enter human clinical trials, being able understand the relationship of pharmacokinetics and target engagement with clinical biomarkers and efficacy is crucial for attributing the pharmacological effect of the inhibitor to perturbation of protein activity.

Although target engagement biomarkers for biopharmaceutical drugs have become routine for linking the plasma pharmacokinetics of a drug with its clinical efficacy in humans, this approach has been less commonly used with small‐molecule inhibitors due to the difficulty of measuring drug‐protein interactions in target tissues. For biopharmaceutics, the high‐affinity binding of antibodies to their antigen makes immunoassay a useful tool for measuring target engagement. A recent report on CC‐292, a small‐molecule covalent BTK inhibitor, describes a quantitative translational methodology to measure CC‐292‐BTK engagement by a direct ELISA assay that detects the presence of drug‐free BTK protein.^13^ In this study, we describe a closely similar set of assays for assessing target engagement to RIPK1 protein, an assay we term the TEAR1 assay.

We have previously shown that RIPK1 protein undergoes significant structural alterations in the presence of our highly selective BOAz class of RIPK1 kinase inhibitors as these inhibitors have a different binding mode than other RIPK1 inhibitors.^2^, ^20^, ^21^, ^22^ Taking advantage of these conformational changes in RIPK1 structure, we identified an antibody which fails to bind RIPK1 protein when the BOAz class of RIPK1 inhibitors are present. This FREE‐RIPK1 antibody only recognizes RIPK1 protein in the absence of inhibitor binding. It is thought that upon inhibitor binding to RIPK1 protein, the epitope region recognized by the antibody is no longer available to bind the FREE‐RIPK1 antibody. In parallel, we identified a second promiscuous antibody that binds RIPK1 irrespective of whether the RIPK1 inhibitor is bound or not. This TOTAL‐RIPK1 antibody recognizes an epitope C‐terminal to the FREE‐RIPK1 epitope and is less likely to undergo a conformational change in the presence of this inhibitor series, however, this has yet to be proven using structural studies, as the epitope (amino acid region 300‐450) resides in regions which are not contained in our crystal structure (amino acid region 1‐294). Together, using this set of two immunoassays measuring FREE‐RIPK1 and TOTAL‐RIPK1, the TEAR1 assay monitors the level of target engagement by the BOAz class of RIPK1 inhibitors.

Although the TEAR1 assay is effective for measuring target engagement of RIPK1 protein by BOAz inhibitors, it is not currently applicable to every chemotype of RIPK1 small‐molecule inhibitors. As demonstrated using HDX‐MS, a more traditional hinge‐binding kinase inhibitor, GSK'064, does not induce changes in solvent accessibility in the RIPK1 activation loop to the extent observed for GSK'481. As such, GSK'064 does not prevent FREE‐RIPK1 antibody binding to RIPK1 protein in the TEAR1 assay, despite inhibiting RIPK1 kinase activity.^20^ Similarly, necrostatin‐type RIPK1 kinase inhibitors also fail to prevent FREE‐RIPK1 antibody binding in the TEAR1 assay (data not shown). Even though this assay is not applicable for these other classes of inhibitors, it is believed that this methodology would be applicable to other drug discovery programs. A combination of HDX‐MS and cocrystal structures may help to identify potential regions for driving the design of possible target engagement antibodies for use with these molecules or other programs in the future.

The TEAR1 assay has been used successfully with the lead RIPK1 clinical candidate, GSK2982772, and a number of other tool RIPK1 inhibitors of the same chemical class during preclinical development to assess target engagement in cells and tissues. In a nonhuman primate model, RIPK1 target engagement was measured in multiple tissues and correlated well with predicted levels of target engagement based on measured drug levels.

While our experiments to date have shown good correlation between predicted (ie, PK drug levels) and measured (ie, TEAR1 assay) target engagement, we have yet to explore whether this correlation holds in disease settings. It is known that RIPK1 can become extensively modified by phosphorylation and ubiquitination following activation of upstream signaling pathways^8^; ^30^ and these modifications could change either the affinity of inhibitors for RIPK1 or the ability of the TEAR1 assay antibodies to detect RIPK1.

Moving forward, the TEAR1 assay will be used in early clinical development to validate its utility to monitor target engagement in blood during phase 1 studies and to monitor target engagement in disease‐relevant target tissues in future phase 2/3 proof‐of mechanism and proof‐of‐concept studies. Having the ability to quantify target engagement in these studies using the TEAR1 assay will be invaluable in interpreting clinical studies with these novel RIPK1 inhibitors and understanding the relationship between target engagement, pathway inhibition, and clinical response.

## DISCLOSURE

The authors declare the following competing financial interests: All authors are current employees and stockholders of GlaxoSmithKline.

## AUTHOR CONTRIBUTIONS

Participated in research design: Finger, Campobasso, Deutsch, Lich, Maguire, Nagilla, Votta, Gough. Conducted experiments: Finger, Brusq, Campobasso, Cook, Deutsch, Haag, Jenkins, Joglekar, Lich, Maguire, Rivera, Sun. Performed data analysis: Finger, Brusq, Campobasso, Cook, Deutsch, Haag, Harris, Joglekar, Lich, Nagilla, Rivera, Sun, Votta, Gough. Wrote or contributed to the writing of the manuscript: Finger, Campobasso, Maguire, Nagilla, Votta, Bertin, Gough.
