# Supplementary material for: Identification of an antibody‐based immunoassay for measuring direct target binding of RIPK1 inhibitors in cells and tissues
Source: Pharmacol Res Perspect. 2017 Dec 6;5(6):e00377. doi: 10.1002/prp2.377 (PMC5723705; doi:10.1002/prp2.377)
Supplement: Supplementary file 1 [file PRP2-5-e00377-s001.pdf]

Supplemental Table 1: RIPK1 antibodies for screening RIPK1 target engagement

A

Antibody	Vendor	Catalog #	Epitope*	Host Species	pAb/mAb
Ab1	Abcam	ab106393	N-terminal	Rabbit	pAb
Ab2	Abcam	ab137451	165-402	Rabbit	pAb
Ab3	Abcam	ab125072	300-450	Rabbit	pAb
Ab4	Abcam	ab2035	420-433	Rabbit	mAb
Ab5	Cell Signaling	3493	L190 +/-10-15aa	Rabbit	mAb
Ab6	Cell Signaling	4926	R413 +/- 10-15aa	Rabbit	pAb
Ab7	Santa Cruz	SC-7881	465-671	Rabbit	pAb

* Epitope reported in datasheet

Supplemental Figure 1: Structures of RIPK1 inhibitors

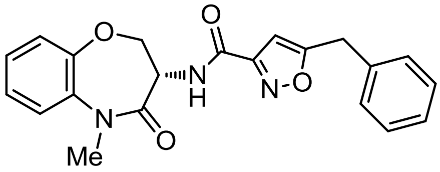

GSK2882481

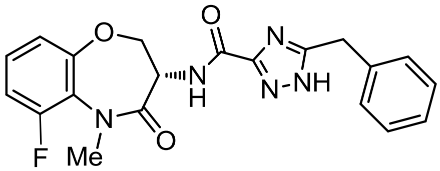

GSK3011253

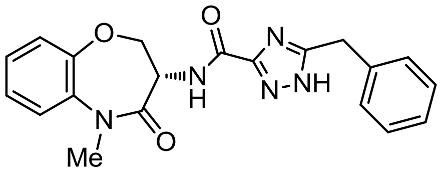

GSK2982772

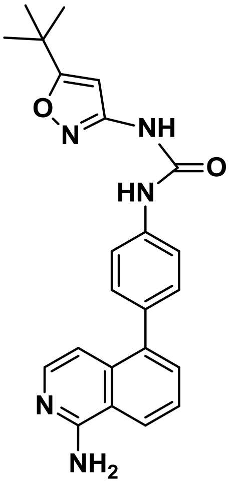

GSK2267064

Supplemental Figure 2: RIPK1 target engagement in HT-29 cells using the TEAR1 assay

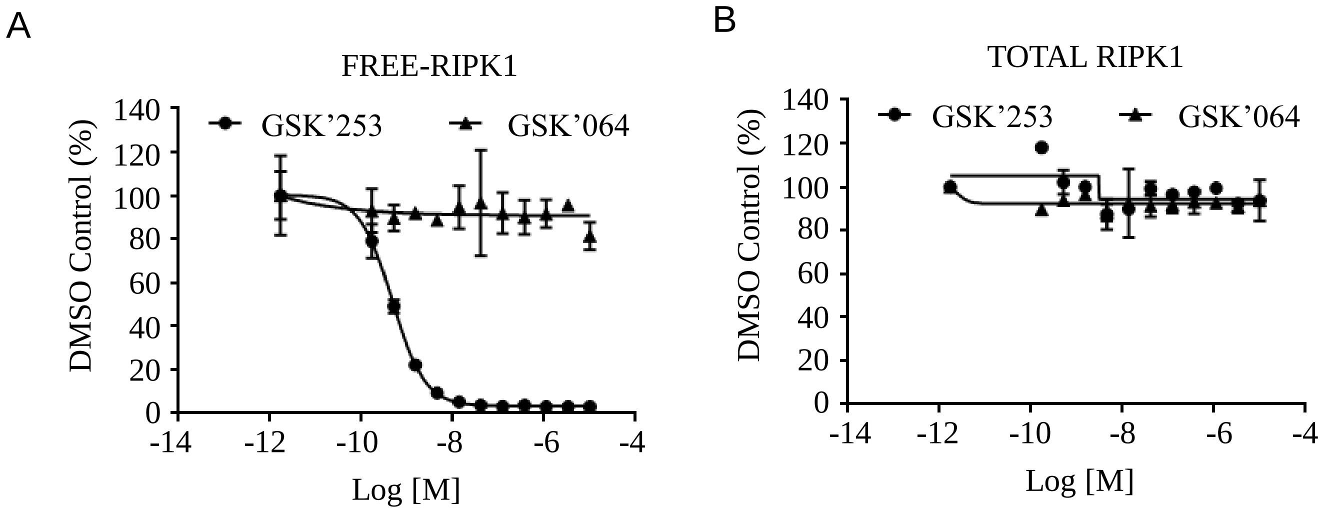

Supplemental Table 2: Blood PK of GSK'253 in male cynomolgus monkeys

Animal ID	Time (hr)	Conc. (ng/mL)	Mean Conc. (ng/mL ± SD)	Predicted TE (%)	Mean Predicted (% ± SD)	Observed TE (%)	Mean Observed (% ± SD)
3C4-20	4	143.48	197.1 ± 72.4	97.9	98.3 ± 0.6	93.8	96.3 ± 2.1
129-101	4	276.7		98.9		92.3	
129-116	4	141.37		97.9		97.7	
M05834	4	285.05		98.9		97.9	
124-767	4	163.26		98.1		96.7	
M01240	4	124.71		97.6		96.9	
M05802	4	153.53		98.0		97.8	
M05814	4	288.3		98.9		97.4	
129-116	19	8.86	9.1 ± 4.9	74.1	71.4 ± 9.5	90.4	82.7 ± 6.5
M05834	19	5.27		63.0		84.5	
124-767	19	11.78		79.2		82.0	
M01240	19	6.04		66.1		72.5	
M05802	19	4.93		61.4		78.7	
M05814	19	17.46		84.9		87.8	
124-767	30	3.95	3.5 ± 1.2	56.0	51.2 ± 10.8	63.2	53.5 ± 10.1
M01240	30	1.67		35.0		42.8	
M05802	30	4.17		57.4		47.0	
M05814	30	4.01		56.4		61.0	
M05802	48	0.79	0.5 ± 0.3	20.3	14.6 ± 8.1	18.3	18.4 ± 0.2
M05814	48	0.3		8.8		18.6	

Supplemental Table 3: Skin PK and TE of GSK'253 in male cynomolgus monkeys

Animal ID	Time (hr)	Conc. (ng/mL)	Mean Conc. (ng/mL ± SD)	Predicted TE (%)	Mean Predicted (% ± SD)	Observed TE (%)	Mean Observed (% ± SD)	Predicted-Observed (%)	Mean Difference (% ± SD)
3C4-20	4	116.6	107.9 ± 38.8	97.4	96.9 ± 1.0	75.2	76.5 ± 8.2	22.2	20.4 ± 8.9
129-101	4	176.1		98.3		60.4		37.9	
129-116	4	89.9		96.7		82.5		14.2	
M05834	4	152.1		98.0		77.6		20.4	
124-767	4	67.0		95.6		88.4		7.2	
M01240	4	68.2		95.7		74.6		21.0	
M05802	4	93.8		96.8		80.7		16.1	
M05814	4	99.1		97.0		72.8		24.1	
129-116	19	10.7	9.4 ± 1.4	77.5	74.9 ± 3.0	49.9	62.4 ± 11.2	27.7	12.5 ± 13.5
M05834	19	9.0		74.3		64.7		9.6	
124-767	19	8.8		74.0		74.6		-0.6	
M01240	19	7.0		69.4		68.8		0.6	
M05802	19	9.7		75.9		50.2		25.7	
M05814	19	11.3		78.5		66.4		12.1	
124-767	30	5.4	3.3 ± 1.4	63.5	50.4 ± 8.9	44.4	47.3 ± 4.3	19.1	3.1 ± 11.2
M01240	30	2.8		47.2		53.7		-6.5	
M05802	30	2.4		44.1		46.3		-2.2	
M05814	30	2.7		46.7		44.9		1.9	
M05802	48	0.5	1.2 ± 1.0	13.2	25.8 ± 17.9	14.7	34.4 ± 27.9	-1.5	-8.6 ± 10.1
M05814	48	1.9		38.5		54.2		-15.7	

Supplemental Table 4: Colon PK and TE of GSK'253 in male cynomolgus monkeys

Colon Tissue

Animal ID	Time (hr)	Concentration (ng/mL)	Predicted TE (%)	Observed TE (%)
3C4-20	4	40.2	92.7	86.9
129-101	4	28.3	90.0	88.5
129-116	19	7.2	69.5	92.5
M05834	19	17.4	84.7	82.4
124-767	30	3.6	53.6	55.6
M01240	30	8.1	72.1	23.4
M05802	48	4.2	57.5	1.8
M05814	48	1.9	37.2	64.6

Supplemental Table 5: Synovium PK and TE of GSK'253 in male cynomolgus monkeys

Synovium Tissue

Animal ID	Time (hr)	Concentration (ng/mL)	Predicted TE (%)	Observed TE (%)
3C4-20	4	9.8	75.8	85.8
129-101	4	24.3	88.6	87.2
129-116	19	1.2	26.9	83.2
M05834	19	1.2	28.1	58.7
124-767	30	1.2	28.4	56.1
M01240	30	1.6	33.1	41.6
M05802	48	1.0	24.5	5.8
M05814	48	0.7	18.2	13.7
